# RBM15 enhances paclitaxel resistance in triple-negative breast cancer by targeting m^6^A methylation of TNFSF9 and inducing polarization of tumor-associated macrophages to M2 phenotype

**DOI:** 10.1186/s41065-025-00534-0

**Published:** 2025-08-19

**Authors:** Jinkun Fu, Chao Wei, Yijian Chen, Xiaoming He, Kun Zhang

**Affiliations:** 1 Department of General Surgery, Xianyang Hospital of Yan’an University, Xianyang, Shaanxi China; 2 Department of Tumor and Thoracic Surgery, Xianyang Hospital of Yan’an University, No. 38 Wenlin Road, Xianyang, Shaanxi 712000 China

**Keywords:** Triple-negative breast cancer, PTX-resistant, TNFSF9, RBM15, M2 polarization

## Abstract

**Background:**

Triple-negative breast cancer (TNBC) is one of the breast cancer subtypes with a poor prognosis, and the current main treatment modalities include surgical resection and adjuvant chemotherapy. However, the development of drug resistance in tumor cells to chemotherapeutic agents poses great challenges to anticancer treatment.

**Methods:**

Bioinformatics analysis was used to screen the up-regulated genes in paclitaxel (PTX)-resistant TNBC cells. Cell viability was measured by a CCK-8 kit. TNFSF9 (Tumor necrosis factor receptor superfamily member 9) protein level was detected by Western blot (WB) assay. PTX-resistant TNBC cell lines (MDA-MB-231/PTX, MDA-MB-468/PTX) were constructed and their drug resistance was shown by IC50. The EdU, flow cytometry, Transwell, and other commercial kits were applied to detect the proliferation, apoptosis, migration, invasion, macrophage M2 polarization, and glycolysis of PTX-resistant TNBC cells. RBM15 (RNA binding motif protein 15) levels were measured by RT-qPCR and WB assays. The RIP, MeRIP, and actinomycin D assays were used to analyze the interaction between TNFSF9 and RBM15. The effect of RBM15/TNFSF9 on PTX sensitivity in vivo was verified by xenograft tumor experiments.

**Results:**

TNFSF9 was highly expressed in PTX-resistant TNBC cells. Silencing of TNFSF9 enhanced the sensitivity to PTX. Silencing TNFSF9 induced polarization of macrophages from M2 to M1 phenotype and the release of IL-1β and TNF-α, but decreased the levels of IL-10 and TGF-β. RBM15 targeted the N6-adenylate methylation (m^6^A) modification of TNFSF9, and overexpression of TNFSF9 could reverse the tumor-suppressing effect of silencing RBM15 on PTX-resistant TNBC cells *in vitro* and transplanted tumors in vivo. Samples from PTX-sensitive and PTX-resistant TNBC patients proved that RBM15 regulated TNFSF9’s high expression in PTX-resistant TNBC tissues.

**Conclusion:**

This study demonstrated that RBM15 enhanced PTX resistance in TNBC by promoting m^6^A methylation in TNFSF9 and inducing M2 polarization of tumor-associated macrophages.

**Graphical abstract:**

**Figure:**
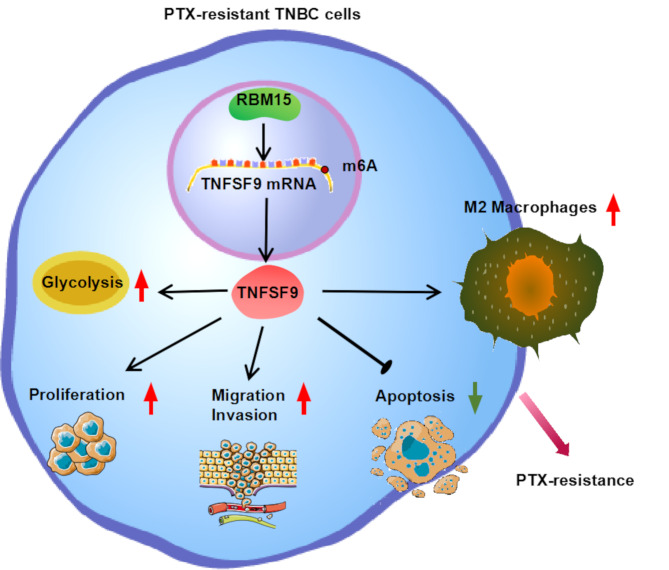
RBM15/TNFSF9 enhances the resistance of TNBC cells to PTX. RBM15 induced m^6^A modification of TNFSF9 to stabilize its expression by binding to it. Subsequently, high levels of TNFSF9 enhanced the proliferation, migration, invasion, and glycolytic pathways of PTX-resistant TNBC cells, but blocked the occurrence of apoptosis. At the same time, the M2 phenotype polarization of macrophages was also promoted. All these effects enhanced the resistance of TNBC cells to PTX

**Supplementary Information:**

The online version contains supplementary material available at 10.1186/s41065-025-00534-0.

## Highlights

1. RBM15 enhanced TNFSF9 stabilization and induced m^6^A methylation.

2. RBM15 targeted TNFSF9 expression both in vivo and in vitro.

3. RBM15/TNFSF9 induced M2 polarization of tumor-associated macrophages and enhanced paclitaxel resistance in TNBC.

**Supplementary Information:**

The online version contains supplementary material available at 10.1186/s41065-025-00534-0.

## Introduction

Breast cancer is the most common malignant tumor among all races of women [1, 2]. Worldwide, breast cancer is the leading cause of death among cancer patients [3]. Among its subtypes, triple-negative breast cancer (TNBC) is one of the highly aggressive forms [4] representing 10–20% of all breast cancer pathologies. Just because of its high malignancy and poor prognosis, its case-fatality rate ranks second among female malignancies, posing a severe threat to women’s health [5–7]. Chemotherapy is currently the main systemic therapy for treating TNBC. Paclitaxel (PTX), as the primary chemotherapy regimen for patients with early-stage or metastatic TNBC, in combination with immune checkpoint inhibitors, significantly prolonged the survival of patients with advanced TNBC [8, 9]. Unfortunately, TNBC patients often develop resistance to PTX within approximately 5 ~ 7 months after PTX treatment, accelerating the deterioration of their tumors [10, 11]. Hence, it is imperative to promptly identify novel therapeutic targets for PTX resistance in TNBC as soon as possible. 

Tumor necrosis factor superfamily member 9 (TNFSF9) was initially discovered on antigen-presenting cells, such as macrophages and dendritic cells, and its expression level was upregulated during the activation of these cells [12, 13]. Currently, TNFSF9 has been confirmed by numerous reports to be correlated with the occurrence and development of tumors. TNFSF9 exerted oncogenic effects in pancreatic cancer [14] prostate cancer [15] and glioma [16] which also positively correlate with M2 polarization of macrophages. Additionally, in breast cancer, TNFSF9 enhanced the migration of monocytes/macrophages while promoting osteoclast differentiation, thereby accelerating bone metastasis of breast cancer [17]. However, despite the significant attention given to understanding PTX resistance in TNBC, the precise role and underlying mechanisms of TNFSF9 in this context have not been fully elucidated. RNA binding motif protein 15 (RBM15) is an RNA-binding motif containing protein involved in a variety of cellular processes, including mRNA splicing, stability, and translational regulation. In recent years, the role of RBM15 in a variety of cancers has gradually attracted attention, especially in breast cancer. Studies have shown that the expression level of RBM15 in breast cancer tissues is usually higher than that in normal breast tissues. This up-regulation may be related to tumor initiation, progression and drug resistance [18]. In addition, RBM15 is a key regulatory protein of RNA N6-methyladenosine (m^6^A) modification, which acts as a cofactor of the methyltransferase complex and is involved in the m^6^A modification of specific RNA molecules, affecting RNA stability, localization, and translation [19]. Therefore, it was speculated that RBM15 is highly likely to be involved in regulating TNFSF9 expression.

Macrophages, which are immune cells, are found widely distributed throughout the body, in both the blood and tissues. In adipose tissue, the accumulation of macrophages is associated with obesity [20]; similarly, the process by which mature macrophages, under the stimulation of a specific microenvironment, develop into different functional phenotypes is known as macrophage polarization [21]. The two phenotypes of macrophage polarization are traditionally activated or inflammatory (M1) and alternatively activated or anti-inflammatory (M2) macrophages, respectively [22, 23]. It is reported that under the influence of the complex tumor microenvironment (TME), macrophages are recruited to the tumor area and polarized into either the M1 state, which suppresses tumor growth, or the M2 state, which enhances tumor growth [24–26]. On this basis, we speculated that TNFSF9 may expedite the malignant progression of TNBC by enhancing the M2 polarization of tumor-associated macrophages (TAMs).

In this research, we evaluated the impact of RBM15/TNFSF9 on the growth and metastasis of PTX-resistant TNBC cells. In addition, we examined the influence of RBM15/TNFSF9 on the polarization state of macrophages within the TNBC tumor microenvironment, further elucidating the molecular mechanisms of PTX resistance in TNBC.

## Materials and methods

### Clinical samples

The tumor tissue samples used in this study were obtained from 35 TNBC patients resistant to PTX and 17 PTX-sensitive patients, all sourced from Xianyang Hospital of Yan’an University during the period from 6 month, 2021 to 12 month, 2024. These participants did not exhibit any symptoms of other diseases or receive any treatment prior to sampling. Written informed consent was obtained from all patients, and our study was approved by the ethics committee of Xianyang Hospital of Yan’an University (Xi an, China).

### Cell culture

Human breast cancer cells MDA-MB-231,MDA-MB-468, and human monocytic leukemia cells THP-1 were bought from the China Center for Type Culture Collection (Wuhan, China) and cultured in Leibovitz’s L-15 medium (Invitrogen, Carlsbad, CA, USA) supplemented with 10% fetal bovine serum (FBS)(Gibco, Grand Island, NY, USA) and 1% penicillin/streptomycin (P/S) (Invitrogen) in a 37℃ incubator without CO_2_ equilibration. THP-1 cells needed to be maintained in the RPMI-1640 medium (Gibco) containing 10% FBS and 1% P/S.

### Construction of PTX-resistant cell lines

MDA-MB-231 and MDA-MB-468 cells were inoculated and the drug resistance was induced by increasing concentration. That is, cells were treated with a lower concentration of PTX at the beginning, and the concentration of paclitaxel was gradually increased as the drug became more adaptive. The concentrations of PTX were 0, 1, 10, 20, 40, and 80 nM. The cell growth and survival rate were monitored in time. When the cells could still maintain a certain proliferation ability under drug treatment, the half maximal inhibitory concentration (IC50) of PTX to PTX-resistant cells was detected by a CCK-8 kit (Beyotime, Shanghai, China). Parental and PTX-resistant cells were seeded in 96-well plates. The parental cells and those resistant to PTX were plated into 96-well plates separately. Once adhered, the cells underwent treatment with PTX for 48 h. Subsequently, the medium was exchanged with one containing 10% CCK-8, and the plates were then incubated in an incubator for 3 h. A microplate reader was used to measure the absorbance at a wavelength of 450 nanometers.

### Cell transfection

To obtain cell lines with stable knockdown of TNFSF9 and RBM15, short hairpin RNA (shRNA) targeting TNFSF9 (sh-TNFSF9), RBM15 (sh-RBM15), and negative control (sh-NC) were constructed and ligated into the pLKO.1 plasmid (GeneChem, Shanghai, China). These constructs were then transfected into the MDA-MB-231/PTX and MDA-MB-468/PTX cell lines using lentiviral vectors. The procedure was as follows: first, the pLKO.1 plasmid (purine-resistant) was linearized, and the annealed shRNA fragment was ligated with the linearized pLKO.1 by T4 DNA ligase (Beyotime). Subsequently, the ligation products were transformed into competent DH5α cells, and the positive clones that were successfully constructed were selected. The pLKO.1-shRNA plasmid was transfected into HEK-293T cells (China Center for Type Culture Collection) in 6-well plate by Lipofectamine 2000 with the help of packaging plasmid psPAX2 (NovoPro, Shanghai, China) and envelope plasmid pMD2.G (NovoPro). After 48 h, viral supernatants were collected to remove cell debris and mixed with complete medium to infect MDA-MB-231/PTX and MDA-MB-468/PTX cell lines at a ratio of 1:4. Finally, Puromycin Dihydrochloride (Beyotime) (1 µg/mL) was used to screen cell lines stably expressing sh-TNFSF9 or sh-RBM15.

The sequence of TNFSF9 was cloned onto the pcDNA3.1 vector (Invitrogen) to construct an overexpression vector. Using the empty vector pcDNA3.1 as the negative control, the TNFSF9 overexpression vector pcDNA3.1-TNFSF9 (oe-TNFSF9) was transiently transfected into MDA-MB-231/PTX and MDA-MB-468/PTX cell lines using Lipofectamine 2000 (Invitrogen).

### Bioinformatics analysis

The genes related to PTX resistance were screened from the GES90564 dataset in the GEO database (https://www.ncbi.nlm.nih.gov/gds) and selected those with significant differential expression at a *P*-value of less than 0.05. Then the Jvenn system was utilized to generate a Venn diagram, where the intersection demonstrates genes that were upregulated in all five PTX-resistant TNBC cell lines.

### Amplification of TNFSF9 and RBM15 genes

To monitor the relative mRNA expression levels of TNFSF9 and RBM15 genes, real-time quantitative PCR (RT-qPCR) assays were performed. According to the manufacturer’s instructions, total RNA in cells was extracted using the Cytoplasmic and Nuclear RNA Purification Kit (Norgen Biotek Corp, Ontario, Canada), and its concentration was determined with the aid of NanoDrop 2000c (Thermo Fisher Scientific, Waltham, MA, USA). Subsequently, with the help of the miScript RT Kit (TaKaRa, Dalian, China), reverse transcription reaction was carried out to obtain the corresponding cDNA. Finally, the cDNA was used as a template for PCR amplification using specific primers on the Real-Time PCR Detection System (Bio-Rad, Shanghai, China). The primers used were as follows: TNFSF9, forward, ‘CTCCGTTTCACTTGCGCTG’, and reverse, ‘TCAGTGTGAAGATGGACGCC’; RBM15, forward, ‘ATGCCTTCCCACCTTGTGAG’, and reverse, ‘GGTCAGCGCCAAGTTTTCTC’; GAPDH, forward, ‘CTTCGCGGGCGACGAT’, and reverse, ‘CCACATAGGAATCCTTCTGACC’. The PCR reaction conditions were 95℃ for 10 s, followed by 45 cycles of 95℃ for 5 s, 60℃ for 10 s, and 72℃ for 10 s. A final extension was performed at 72℃ for 5 min.

### Western blot (WB) assay measurement of protein expression

The expression levels of the tested proteins in the cells were determined by Western blot (WB). In particular, cells were lysed with a protein extraction kit (Beyotime) to extract total proteins, and their concentrations were determined using the Pierce BCA Protein Assay Kit (Thermo Fisher Scientific). The protein lysates were separated on SDS-PAGE and then fully transferred onto polyvinylidene fluoride (PVDF) membrane (GE Healthcare, Piscataway, NJ, USA). Blocking was performed using 5% skimmed milk to prevent nonspecific binding of antibodies to nontarget proteins or other molecules on it. The membrane was then incubated with the primary antibody overnight at 4℃, followed by a two-hour incubation with the secondary antibody. After that, the PVDF membrane was subjected to chemiluminescence detection using the eyoECL Plus Kit (Beyotime). The primary antibodies applied to this study included Anti-4-1BBL (TNFSF9) antibody (1: 1000, ab68185, Abcam, Cambridge, UK), Anti-RBM15 antibody (1: 1000, ab315456, Abcam) and Anti-beta Actin antibody (1:1000, ab8226, Abcam); and the secondary antibodies included HRP conjugated-Goat anti-Rabbit (1:5000, ab205718, Abcam) and HRP conjugated-Goat anti-Mouse (1:5000, ab205719, Abcam).

**Measurement of cell proliferation** EdU assays were employed to evaluate the proliferative capacity of the cells. In brief, cells were seeded in 6-well plates overnight. After corresponding treatments according to the experimental conditions, cells were exposed to EdU working solution (Beyotime) for 2 h. After incubation, the cells were fixed with 4% paraformaldehyde (Beyotime) for 15 min and then treated with 0.3%Triton X-100 (Beyotime) for 10 min to permeabilize. After the above steps, nuclei were stained with 4’,6-diamidino-2-phenylindole (DAPI) (Thermo Fisher Scientific) for another 10 min. At the end, the EdU-labeled cells were visualized and counted using fluorescence microscopy (Olympus, Japan).

**Assessment of apoptosis** Using Annexin V-FITC/PI Apoptosis Detection Kit (Yeasen, Shanghai, China), the effect of TNFSF9 knockdown on apoptosis in PTX-resistant TNBC cells was detected by flow cytometry. After re-suspending the cells in 1×binding buffer (100 µL), they were divided into four groups: an unstained group, a single stained Annexin V (5 µL), a single stained Propidium Iodide (PI) (10 µL), and a double stained Annexin V/PI group for staining avoid light. After 15 min, 1×binding buffer was introduced, and the cells underwent flow cytometry analysis, with fluorescein isothiocyanate (FITC) as the abscissa and PI (PE-A) as the ordinate.

### Monitoring of macrophage polarization

To investigate the effect of silenced TNFSF9 on macrophage polarization, the condition media (CM) from MDA-MB-231/PTX and MDA-MB-468/PTX cells transfected with sh-NC and sh-TNFSF9 were first collected, and co-cultured with THP1-M0 cells to induce their polarization. It should be noted that the THP-1-M0 cells are un-polarized macrophages (M0 type macrophages) derived from THP-1 cells through the induction by phorbol 12, 14-diacetate 13-acetate (PMA) (Beyotime). The cells were then digested with trypsin (Gibco), washed with phosphate buffer solution (PBS), and re-suspended. Anti-human- inducible nitric oxide synthase (iNOS) (Proteintech, Wuhan, China) was added, and the cells were stained avoiding light at 4℃ for 30 min. Following rinsing with PBS, the cells were analyzed for the THP1-M1 phenotypic cells (M1 type macrophages) using flow cytometry. For THP1-M2 phenotypic (M2 type macrophages) analysis, the digested cells were fixed for 20 min, washed with permeabilization buffer, re-suspended, stained with anti-human-CD206 (Proteintech) in the dark at 4 °C for 30 min, washed, re-suspended again, and analyzed by flow cytometry.

### Cell migration and invasion assays

When assessing the migratory capacity of breast cancer cells, MDA-MB-231/PTX and MDA-MB-468/PTX cells were first subjected to serum starvation in serum-free medium for 24 h. Next, complete medium containing 10% FBS (Gibco) was added to the lower chamber of a Transwell plate (Corning, Tewksbury, MA, USA). Subsequently, the cells resuspended in serum-free medium (Gibco) were evenly distributed in the upper chamber and incubated at 37℃ for 24 h. Afterward, the cells in the lower chamber were washed with PBS (Beyotime), fixed with 4% paraformaldehyde (Beyotime), and stained with crystal violet (Beyotime). Following washing, the cells were counted using an optical microscope. For the assessment of invasion capacity, a layer of Matrigel matrix (Bedford, MA, USA) was pre-added to the upper chamber before the aforementioned steps to mimic the extracellular matrix environment in vivo.

### Assay of glycolysis

To verify the impact of silencing TNFSF9 on the glycolysis pathway in PTX-resistant TNBC cells, changes in the glycolytic pathway were assessed by measuring glucose consumption, lactate production, and ATP content by kits. All kits used for detecting glucose, lactate, and ATP were purchased from Beyotime, and the operations were strictly carried out according to the kit instructions.

### Validation of RBM15 and TNFSF9 binding

The RNA immunoprecipitation (RIP) experiment was utilized to detect the interaction between RBM15 and TNFSF9. PTX-resistant TNBC cells were crosslinked using formaldehyde (Beyotime) before being lysed with RIPA buffer (Beyotime). Rabbit IgG (Proteintech) and anti-RBM15 (Proteintech) were added to form immune complexes, which were then precipitated using Protein A/G magnetic beads (Thermo Fisher). Unbound RNA and proteins were separated through multiple washing steps. After crosslink reversal by proteinase K (Yeasen), RNA was extracted and subjected to RT-qPCR analysis via the miScript RT Kit (TaKaRa) to assess the enrichment of TNFSF9 mRNA. The PCR reaction conditions were 95℃ for 10 s, followed by 45 cycles of 95℃ for 5 s, 60℃ for 10 s, and 72℃ for 10 s. A final extension was performed at 72℃ for 5 min.

### Determination of m^6^A methylation modification levels of TNFSF9’s mRNA

To verify whether RBM15 can regulate the m^6^A methylation modification of TNFSF9 mRNA using the MeRIP-qPCR assay, the following steps were taken. Firstly, total RNA was extracted using Trizol reagent (Thermo Fisher) and then fragmented. Subsequently, anti-m^6^A antibody and rabbit IgG (Proteintech) were co-incubated with the fragmented RNA to form complexes. After precipitating the complexes using Protein A/G beads, the methylated RNA enriched was separated from the interacting proteins. Reverse transcription was performed followed by RT-qPCR via the miScript RT Kit (TaKaRa) to detect the mRNA expression levels. The PCR reaction conditions were 95℃ for 10 s, followed by 45 cycles of 95℃ for 5 s, 60℃ for 10 s, and 72℃ for 10 s. A final extension was performed at 72℃ for 5 min.

### RNA stability assay

To examine the effect of RBM15 on TNFSF9 mRNA expression, cells transfected with sh-NC and sh-RBM15 were first inoculated and treated with actinomycin D. Cells were collected at 0, 4, 8, and 12 h to extract total RNA to remove genomic DNA. Following reverse transcription, RT-qPCR was performed.

**Verification of the role of the RBM15/TNFSF9 axis** **in vivo.** To further investigate the impact of RBM15/TNFSF9 on the sensitivity of resistant cells to PTX in vivo, tumor xenograft experiments were conducted on BALB/c nude mice (SLAC, Shanghai, China). MDA-MB-231/PTX cells stably transfected with sh-NC, sh-RBM15, sh-RBM15 + oe-TNFSF9 were suspended at a concentration of 1 × 10^7^ cells per 0.2 mL of PBS: Matrigel (3:1). The mice were divided into four groups, and a TNBC subcutaneous xenograft model was established by subcutaneous injection of 10 µL of cell suspension into the right axilla. When the tumors reached a volume of 100 mm³, PTX (20 mg/kg) (dissolved in PBS as the carrier control) was administered intraperitoneally twice a week. Tumor volumes were monitored in real-time and calculated using the formula: Volume (mm³) = Width² × Length / 2. After three weeks, the mice were euthanized, and the subcutaneous tumor tissues were completely dissected, photographed, and weighed. The mice experiments were approved by the Xianyang Hospital of Yan’an University Animal Protection Committee in accordance with the manual.

### Statistical analysis

For each experimental group, three separate and parallel experiments were conducted, and the data were expressed as the mean ± standard deviation (SD) which was analyzed by GraphPad Prism 7. Flow data were analyzed using FlowJo v10. As required, comparisons across different groups were conducted using either a one-way ANOVA or a Student’s *t*-test. Statistical significance was determined at *P* < 0.05.

## Results

### TNFSF9 is highly expressed in PTX-resistant TNBC cells

**Fig. 1:**
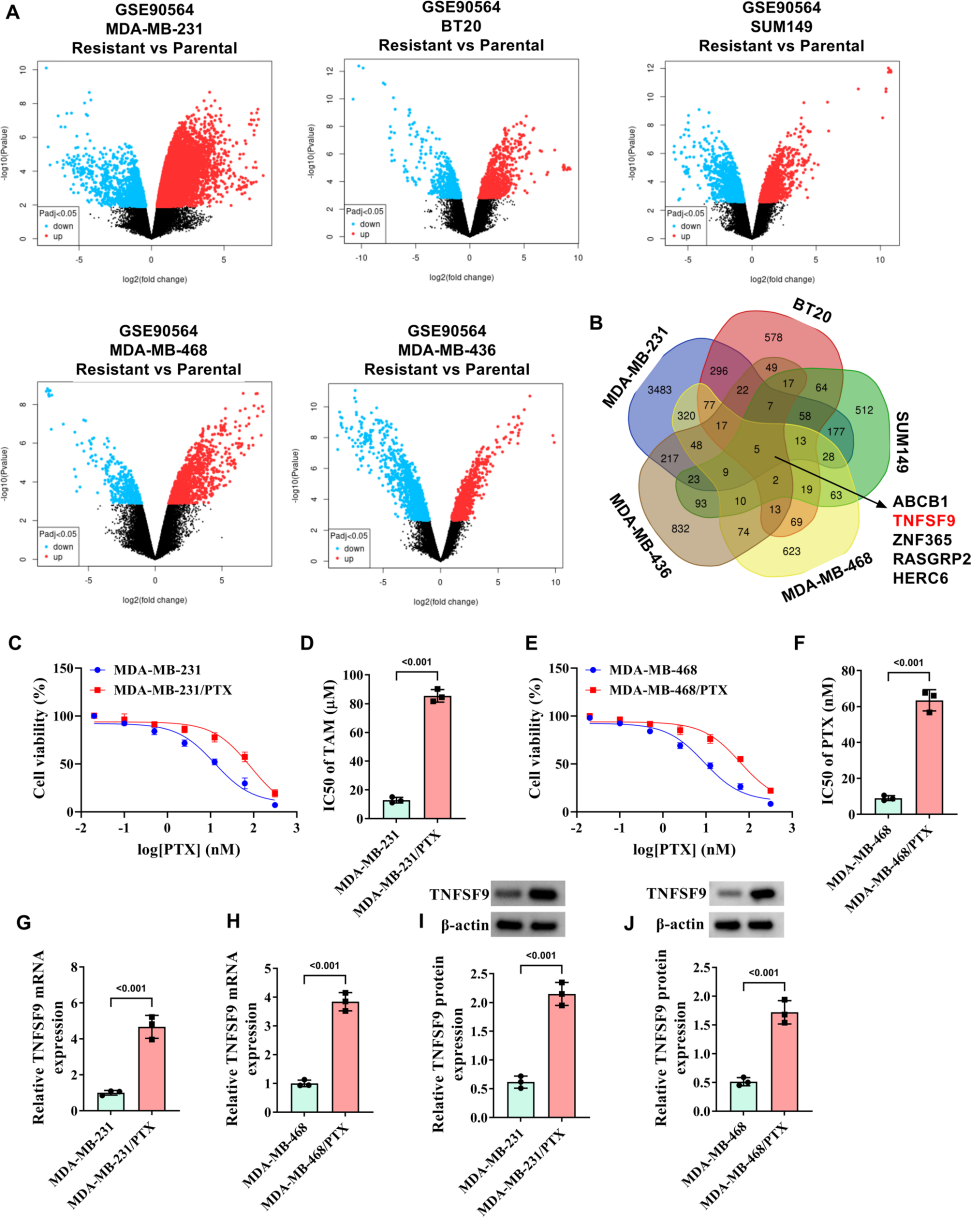
TNFSF9 expression in PTX-resistant TNBC cells. The MDA-MB-231, MDA-MB-468, MDA-MB-231/PTX, and MDA-MB-468/PTX cells were treated with PTX at 5, 10, 15, 20, 25, and 30 µM. (**A**) The volcano plots for DEGs identified using GSE90564 datasets. (**B**) The intersection between the five sets of DEGs was presented by the Venn diagram. (**C**-**F**) The CCK-8 kit was used to detect the viability of parental cells (MDA-MB-231, MDA-MB-468) and PTX-resistant cells (MDA-MB-231/PTX, MDA-MB-468/PTX) as well as the IC50 of PTX. (**G**-**H**) The expression of TNFSF9 mRNA in PTX-resistant cells was detected by RT-qPCR. (**I**-**J**) WB was used to detect the protein expression of TNFSF9 in PTX-resistant cells

The GES90564 dataset was downloaded from the GEO database to analyze differentially expressed genes in PTX-resistant BC cell lines and the results were presented using a volcano plot (Fig. 1A). A Venn diagram was applied to display the genes that were all up-regulated in five types of TNBC PTX-resistant cells (Fig. 1B), including the TNFSF9 gene. Subsequently, CCK-8 assay results of PTX-resistant cell lines established based on MDA-MB-231 and MDA-MB-468 cells associated with TNBC showed that when the treatment concentration of PTX was between 40 nM and 80 nM, the survival rate of MDA-MB-231/PTX and MDA-MB-468/PTX cell lines could reach about 50%, while the survival rate of parental cells MDA-MB-231 and MDA-MB-468 was only about 25%, indicating that drug-resistant cells were more tolerant to PTX (Fig. 1C-F). In addition, both WB and RT-qPCR assay results confirmed that TNFSF9 was highly expressed in PTX-resistant cells in contrast to parental cells (Fig. 1G-J). In conclusion, this part of the results identified a high level of TNFSF9 expression in PTC-resistant TNBC cells, indicating that TNFSF9 expression might be associated with the development of TNBC. More importantly, in Supplementary Fig. 1, it was found that the expression of M2-type macrophage marker (CD206) was significantly higher and the expression of M1-type macrophage marker (iNOS) was significantly lower in tumor tissues of PTX-resistant patients than those of PTX-sensitive patients. This suggested that the development of PTX resistance in TNBC cells may be related to the M2 polarization of macrophages.

### Knockdown of TNFSF9 enhances the sensitivity of PTX-resistant TNBC cells to PTX

**Fig. 2:**
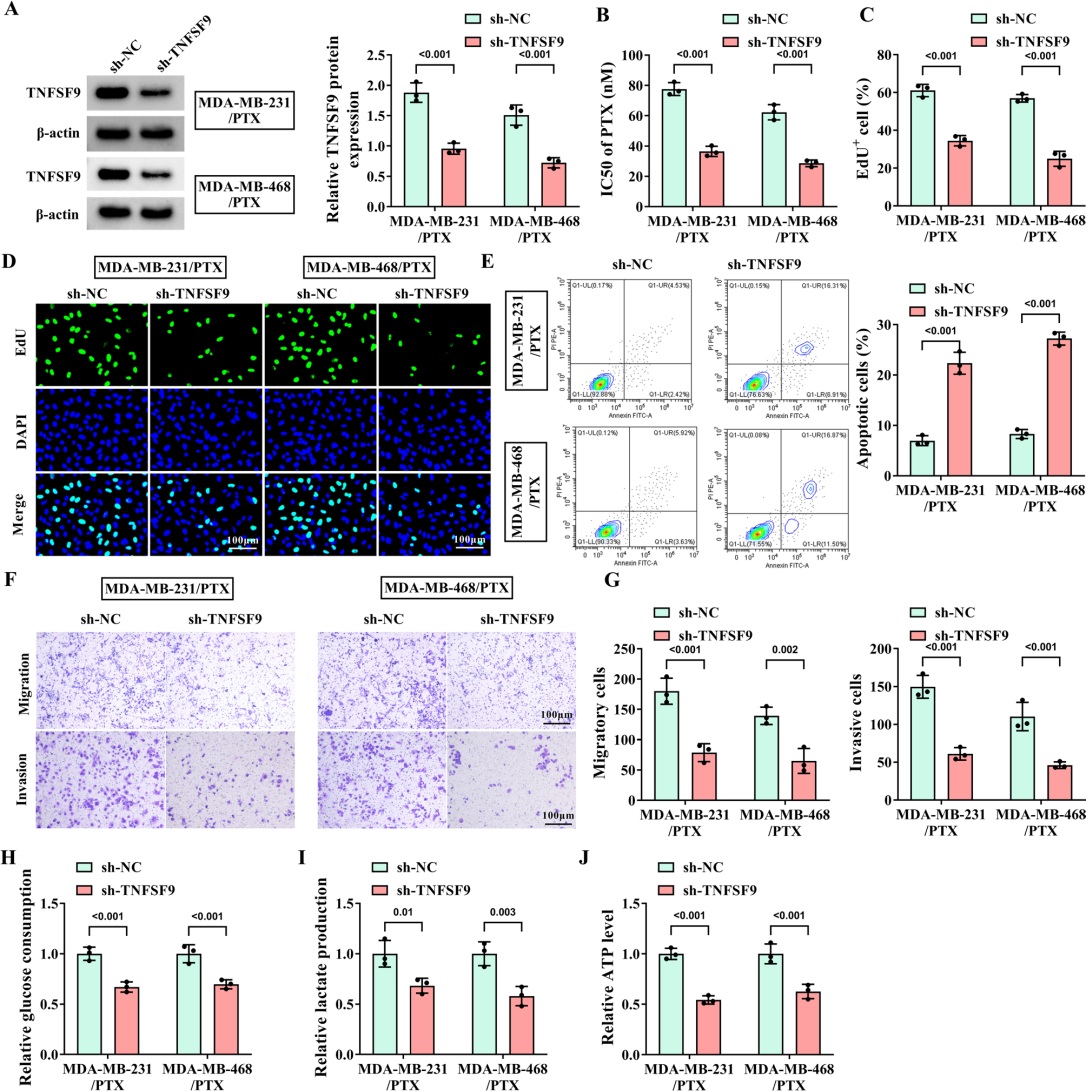
Effect of knockdown of TNFSF9 on the progression of PTX-resistant TNBC cells. The MDA-MB-231/PTX and MDA-MB-468/PTX cells were transfected with sh-NC and sh-TNFSF9. (**A**) WB was used to detect the knockdown efficiency of PTX-resistant TNBC cell lines with knockdown of TNFSF9. (**B**) The IC50 of PTX was measured by CCK-8 assay. (**C**-**D**) The effect of TNFSF9 knockdown on the proliferation of PTX-resistant cells was determined by EDU assay. (**E**) Flow cytometry was applied to detect the effect of TNFSF9 knockdown on the apoptosis of PTX-resistant cells. (**F**-**G**) Transwell assay was applied to determine the effect of TNFSF9 knockdown on the migration and invasion of PTX-resistant cells. (**H**-**J**) Commercial kits were used to detect the effects of TNFSF9 knockdown on glucose consumption, lactate production and ATP level.

To explore the exact role of TNFSF9 in PTX-resistant cells, TNFSF9 was treated with loss of function. WB analysis was first conducted to verify the stable downregulation of TNFSF9 in PTX-resistant cell lines after TNFSF9 knockdown (Fig. 2A). Fig. 2B displayed that the IC50 of PTX was decreased with the knockdown of TNFSF9 in MDA-MB-231/PTX and MDA-MB-468/PTX cells. Simultaneously, it was clarified that downregulation of TNFSF9 effectively suppressed the proliferation of PTX-resistant cells, but induced their apoptosis (Fig. 2C-E), and remarkably blocked the migration and invasion behaviors of two PTX-resistant cell lines (Fig. 2F-G). Furthermore, we also found that glucose consumption, lactate production, and ATP level were obviously impaired after silencing TNFSF9, indicating that knockdown of TNFSF9 hindered the glycolysis in two PTX-resistant cell lines (Fig. 2H-J). The above evidence suggested that TNFSF9 enhanced the resistance of TNBC cells to PTX.

### PTX-resistant TNBC cells with TNFSF9 knockdown impair M2 polarization of macrophages

**Fig. 3:**
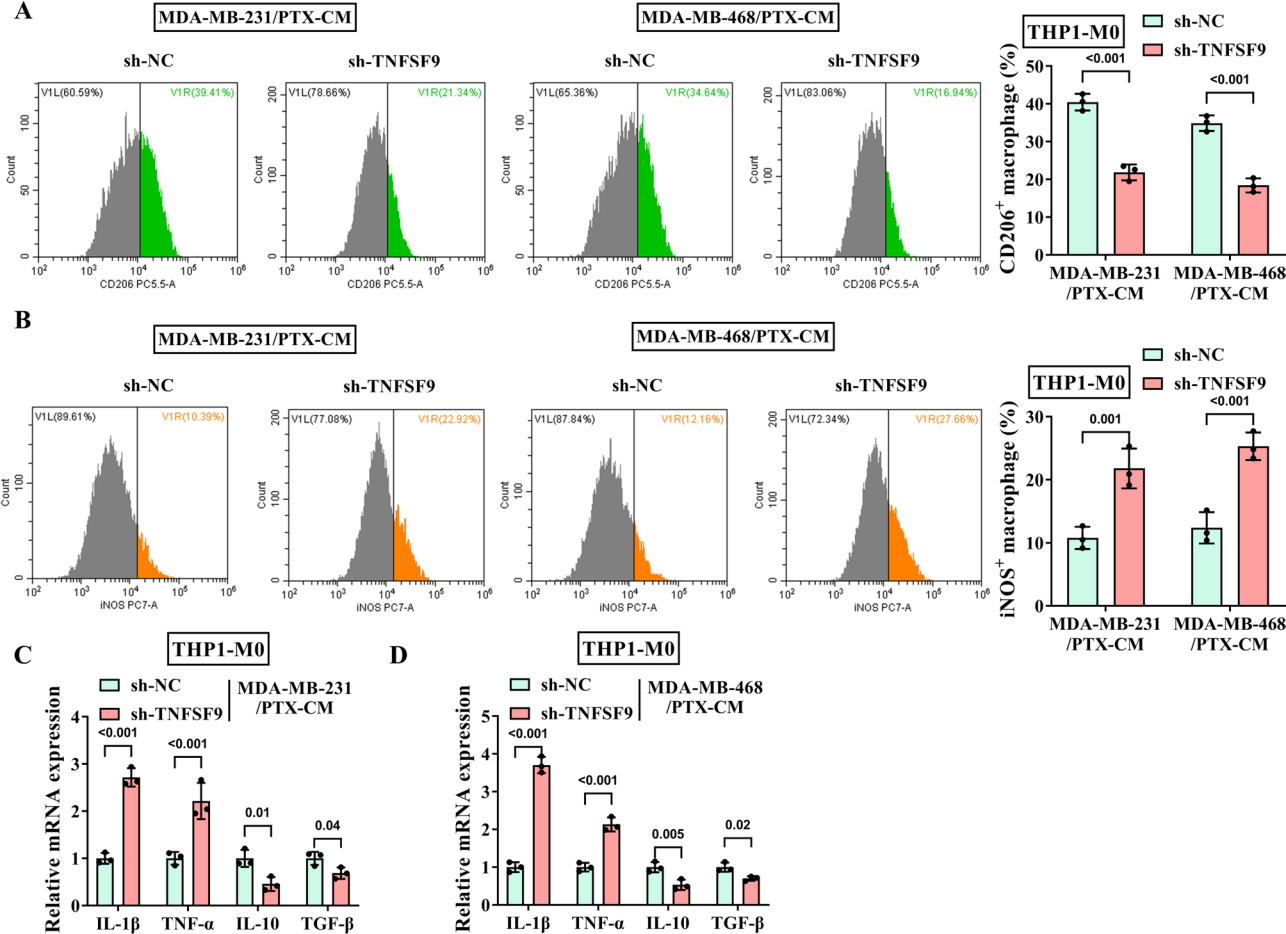
Effect of silencing TNFSF9 on macrophage polarization. The MDA-MB-231/PTX and MDA-MB-468/PTX cells were transfected with sh-NC and sh-TNFSF9. (**A**-**B**) The expression of M2 marker CD206 and M1 marker INOS in THP1-M0 cells were detected by flow cytometry. (**C**-**D**) RT-qPCR was used to measure the expression of M2 marker IL-10 and TGF-β, M1 markers IL-1β and TNF-α

The THP1 cells were induced into macrophage-like THP1-M0 cells and TNBC cell-associated macrophages with TNFSF9 knockdown were constructed. Flow cytometry results demonstrated that the expression of M2 phenotypic marker CD206 in THP1-M0 cells was greatly retarded, while the M1 phenotypic marker INOS level was increased (Fig. 3A-B). Consistent with the above results, the mRNA levels of M2-related interleukin 10 (IL-10) and transforming growth factor-β (TGF-β) were robustly downregulated, but the trend for the M1 marker interleukin 1β (IL-1β) and tumor necrosis factor-α (TNF-α) was opposite (Fig. 3C-D). It was evident that TNFSF9 accelerated the malignant progression of TNBC by facilitating macrophage M2 polarization.

### RBM15 increases the stability of TNFSF9 through m^6^A modification

**Fig. 4:**
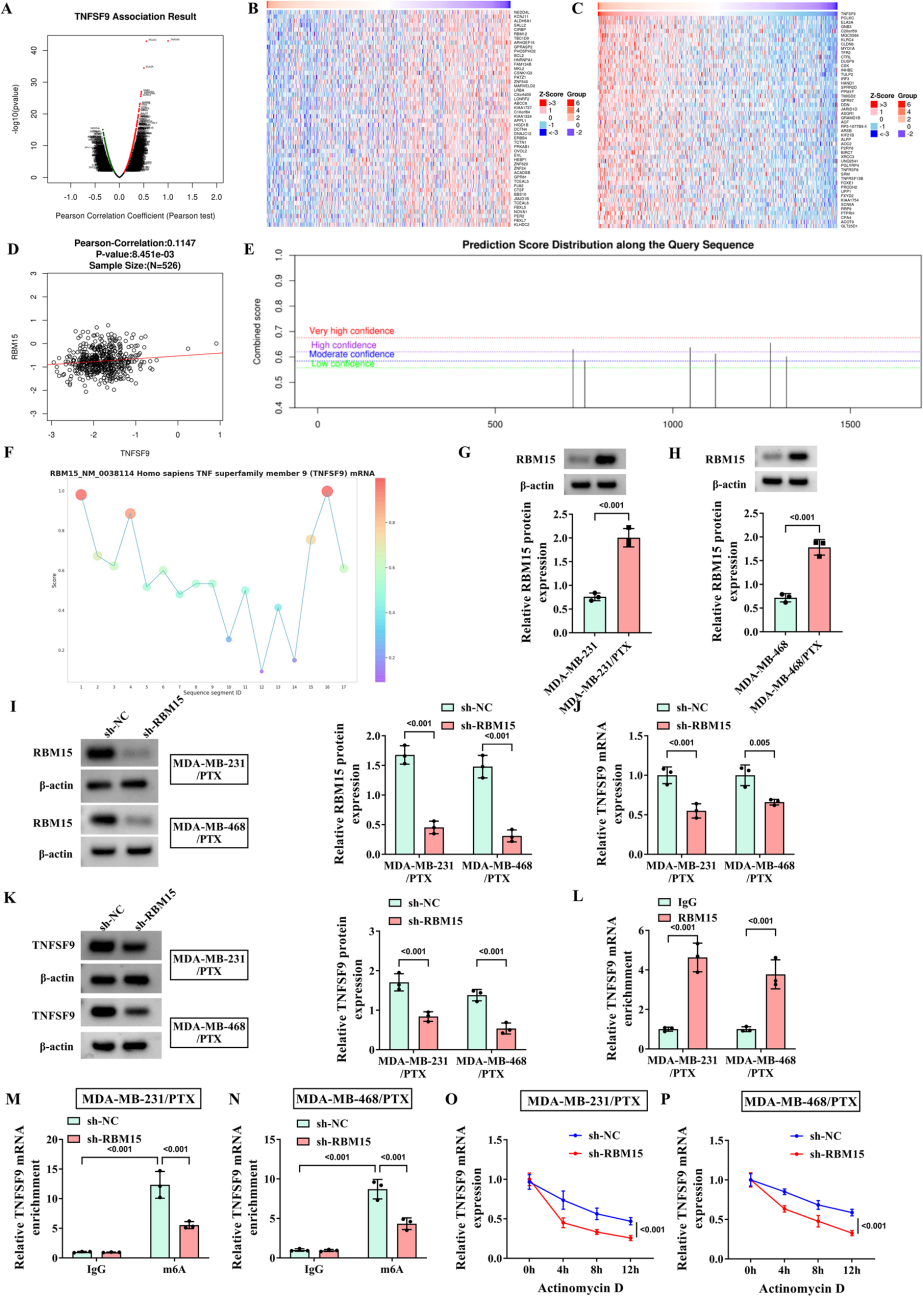
RBM15 increases the stability of TNFSF9 through m^6^A modification. The MDA-MB-231/PTX and MDA-MB-468/PTX cells were transfected with sh-NC and sh-RBM15. (**A**) Genes associated with TNFSF9 in BRCA screened by the linkedomics website. (**B**) Genes positively associated with TNFSF9 in BRCA screened by the linkedomics website. (**C**) Genes negatively associated with TNFSF9 in BRCA screened by the linkedomics website. (**D**) The correlation between TNFSF9 and RBM15 was analyzed by Pearson correlation coefficient. (**E**) An m^6^A site on the mRNA of TNFSF9 was predicted by SRAMP website. (**F**) The potential binding sites of TNFSF9 and RBM15 were predicted by RBP suite website. (**G**-**H**) WB was used to verify the expression of RBM15 in PTX-resistant cells. (**I**) WB was applied to detect the protein level of RBM15 in cells with RBM15 knocked down. (**J**-**K**) Protein and mRNA levels of TNFSF9 in PTX-resistant cells with knockdown of RBM15 were detected by WB and RT-qPCR assay. (**L**) The interaction between RBM15 and TNFSF9 was validated through RIP assay. (**M**-**N**) The MeRIP assay was used to explore the effect of RBM15 on m^6^A modification of TNFSF9. (**O**-**P**) The effect of RBM15 on TNFSF9 mRNA stability was determined by actinomycin D

The volcano plot shown in Fig. 4A presented the genes related to TNFSF9 in breast cancer susceptibility gene (BRCA), which were screened through the Linkedomics website (https://www.linkedomics.org/login.php). And the genes that were positively (Fig. 4B) and negatively (Fig. 4C) correlated with TNFSF9 were shown by heatmap. Based on this information, a certain positive correlation was found between the mRNA of TNFSF9 and RBM15 (Fig. 4D). Interestingly, through the SRAMP website (http://www.cuilab.cn/sramp/), it was predicted that there were m^6^A binding sites on the mRNA sequence of TNFSF9 (Fig. 4E), and the RBPsuite (http://www.csbio.sjtu.edu.cn/bioinf/RBPsuite/) also revealed potential binding sites between TNFSF9 and RBM15 (Fig. 4F). The high expression of PBM15 in PTX-resistant TNBC cells and its stable downregulation in the sh-RBM15 cell lines had been confirmed by WB analysis (Fig. 4G-I). At the same time, the mRNA and protein expression levels of TNFSF9 also exceptionally decreased in both MDA-MB-231/PTX and MDA-MB-468/PTX cells (Fig. 4J-K), as demonstrated by relevant analyses. The RIP experiment validated the previous prediction that RBM15 could bind to and interact with the mRNA of TNFSF9 (Fig. 4L). Similarly, the m^6^A modification of TNFSF9 was prevented by silencing RBM15 (Fig. 4M-N). And more importantly, through actinomycin D assay, it was implicated that the stability of TNFSF9 mRNA is indeed regulated by RBM15 (Fig. 4O-P). Taken together, these lines of evidence suggested that RBM15 bound to TNFSF9 and promoted m^6^A modification of TNFSF9 mRNA to stabilize its expression level in cells.

### RBM15 affects the sensitivity of PTX-resistant TNBC cells to PTX by regulating the expression of TNFSF9

**Fig. 5:**
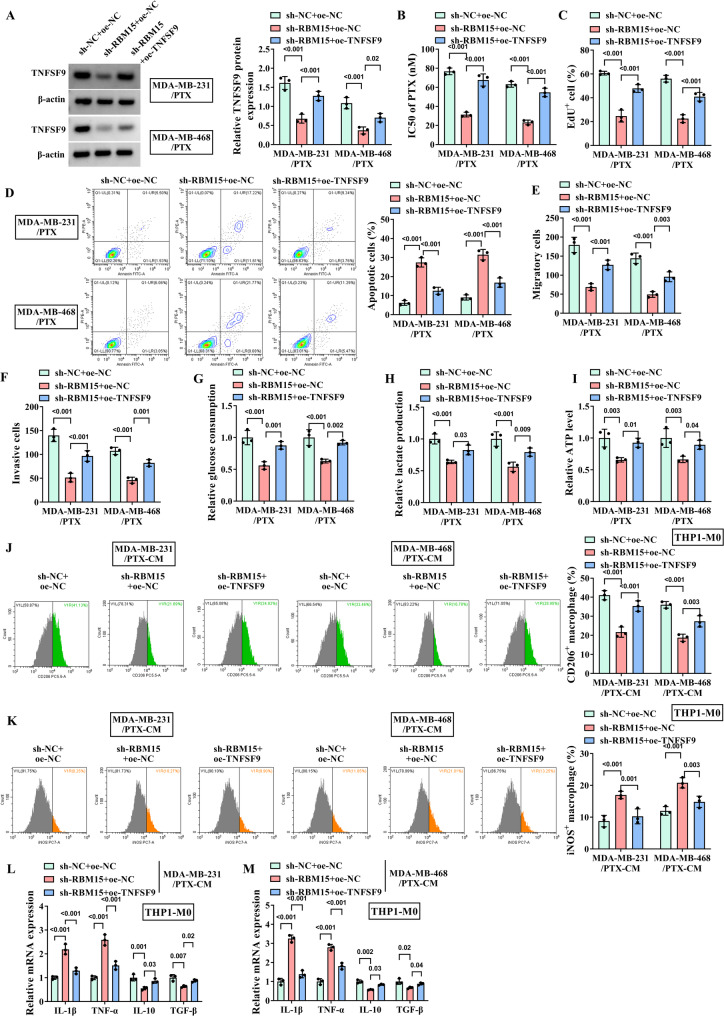
Overexpression of TNFSF9 reverses the effects of RBM15 knockdown. The MDA-MB-231/PTX and MDA-MB-468/PTX cells were transfected with sh-NC + oe-NC, sh-RBM15 + oe-NC, and sh-RBM15 + oe-TNFSF9. (**A**) The overexpression efficiency of sh-RBM15 cell lines transfected with pcDNA-TNFSF9 was determined by WB assay. (**B**) CCK-8 assay was used to detect the IC50 of PTX. (**C**) EdU assay was used to detect the effect of TNFSF9 overexpression on the proliferation of sh-RBM15 cells. (**D**) Flow cytometry was utilized to detect the effect of TNFSF9 overexpression on the apoptosis of sh-RBM15 cells. (**E**-**F**) The effect of TNFSF9 overexpression on the migration and invasion of sh-RBM15 cells were determined by Transwell assay. (**G**-**I**) The commercial kits were used to detect the effect of TNFSF9 overexpression on the glycolytic pathway of sh-RBM15 cells. (**J**-**K**) The expression of M2 marker CD206 and M1 marker INOS were detected by flow cytometry. (**L**-**M**) RT-qPCR was used to measure the expression of IL-10, TGF-β, IL-1β, and TNF-α

Next, the effect of RBM15 and TNFSF9 on the sensitivity of PTX-resistant TNBC cells to PTX was further elucidated by transfusing pcDNA-TNFSF9 into a stable cell line with RBM15 knockdown. WB analysis showed that when RBM15 was silenced, the expression of TNFSF9 was down-regulated (Fig. 5A). As shown in Fig. 5B, after TNFSF9 was overexpressed, the half-maximal inhibitory concentration of PTX for two PTX-resistant cell lines with RBM15 knockdown evidently was increased again. Subsequently, the relevant behaviors of MDA-MB-231/PTX and MDA-MB-468/PTX cells with RBM15 knockdown were analyzed, revealing an effect consistent with that of TNFSF9 silencing in inhibiting the malignant growth and metastasis of PTX-resistant cells. The results of EdU and flow cytometry analysis were shown in Fig. 5C-D. When RBM15 was silenced, the proliferation ability of PTX-resistant cells was remarkably weakened, but the apoptosis rate was greatly increased. Similarly, the migration and invasion behaviors of PTX-resistant cell lines were obviously blocked by knockdown of RBM15 (Fig. 5E-F). Concurrently, further validation through the measurement of glycolysis-related indicators confirmed that transfection of sh-RBM15 in MDA-MB-231/PTX and MDA-MB-468 cells resulted in significant inhibition of intracellular glucose consumption and lactate production, accompanied by a notable decrease in ATP levels (Fig. 5G-I). After that, flow cytometry showed that knockdown of RBM15 reduced the level of CD206 in THP1-M0 cells. On the contrary, the level of iNOS was abnormally increased (Fig. 5J-K). In addition, the mRNA levels of IL-10 and TGF-β were also decreased, while the release of IL-1β and TNF-α were increased (Fig. 5L-M). However, it was interested that all the effects of RBM15 silencing were reversed by overexpression of TNFSF9 in addition to RBM15 knockdown. In other words, TNFSF9 was regulated by RBM15, which together enhanced the resistance of TNBC cells to PTX.

### RBM15 modulates the sensitivity of PTX-resistant TNBC cells to PTX by regulating the expression of TNFSF9 in vivo

**Fig. 6:**
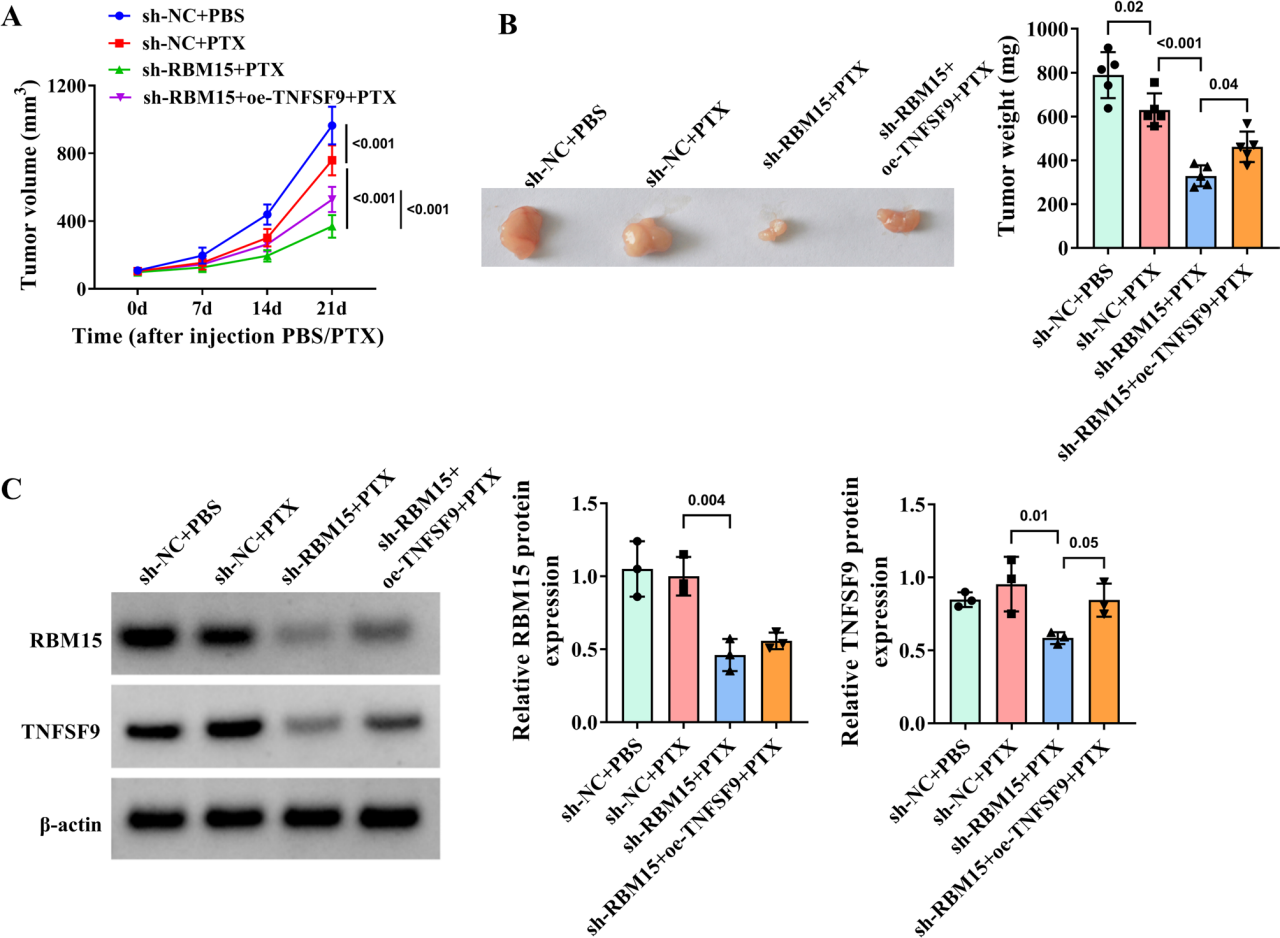
Effect of RBM15/TNFSF9 on the sensitivity of TNBC to PTX in vivo. Mice were injected with MDA-MB-231/PTX cells stably transfected with sh-NC, sh-RBM15, and sh-RBM15 + oe-TNFSF9 and randomly divided into the control (PBS) group and the PTX (20 mg/kg) treatment group. (**A**) The growth rate of xenograft tumors in mice (*n* = 5). (**B**) The weight of tumors after 21 days (*n* = 5). (**C**) WB was used to measure RBM15 and TNFSF9 protein levels in sh-RBM15 cells overexpressing TNFSF9

The xenograft tumor models in nude mice were established to verify the impact of RBM15/TNFSF9 on the sensitivity of PTX-resistant TNBC cells to PTX in vivo. Through real-time monitoring of tumors, we observed that the growth rate, volume and weight of tumors in mice transfected with sh-RBM15 and treated with PTX (20 mg/kg) were distinctly reduced compared to those in the control group of mice. However, in the mice group with overexpression of TNFSF9 in the stably silenced RBM15 cell line, despite PTX treatment, the tumor growth rate, and weight still increased (Fig. 6A-B). Additionally, WB analysis demonstrated that when RBM15 was silenced in MDA-MB-231/PTX cells, the protein level of RBM15 was down-regulated in mouse tumor tissues, accompanied by a concomitant decrease in TNFSF9 levels. However, after silencing RBM15 and then overexpressing TNFSF9, the protein level of TNFSF9 in tumor tissues showed a significant rebound trend (Fig. 6C), confirming that RBM15 could also targeted and regulated TNFSF9 in vivo.

### Both RBM15 and TNFSF9 are highly expressed in tissues of patients with PTX-resistant TNBC

**Fig. 7:**
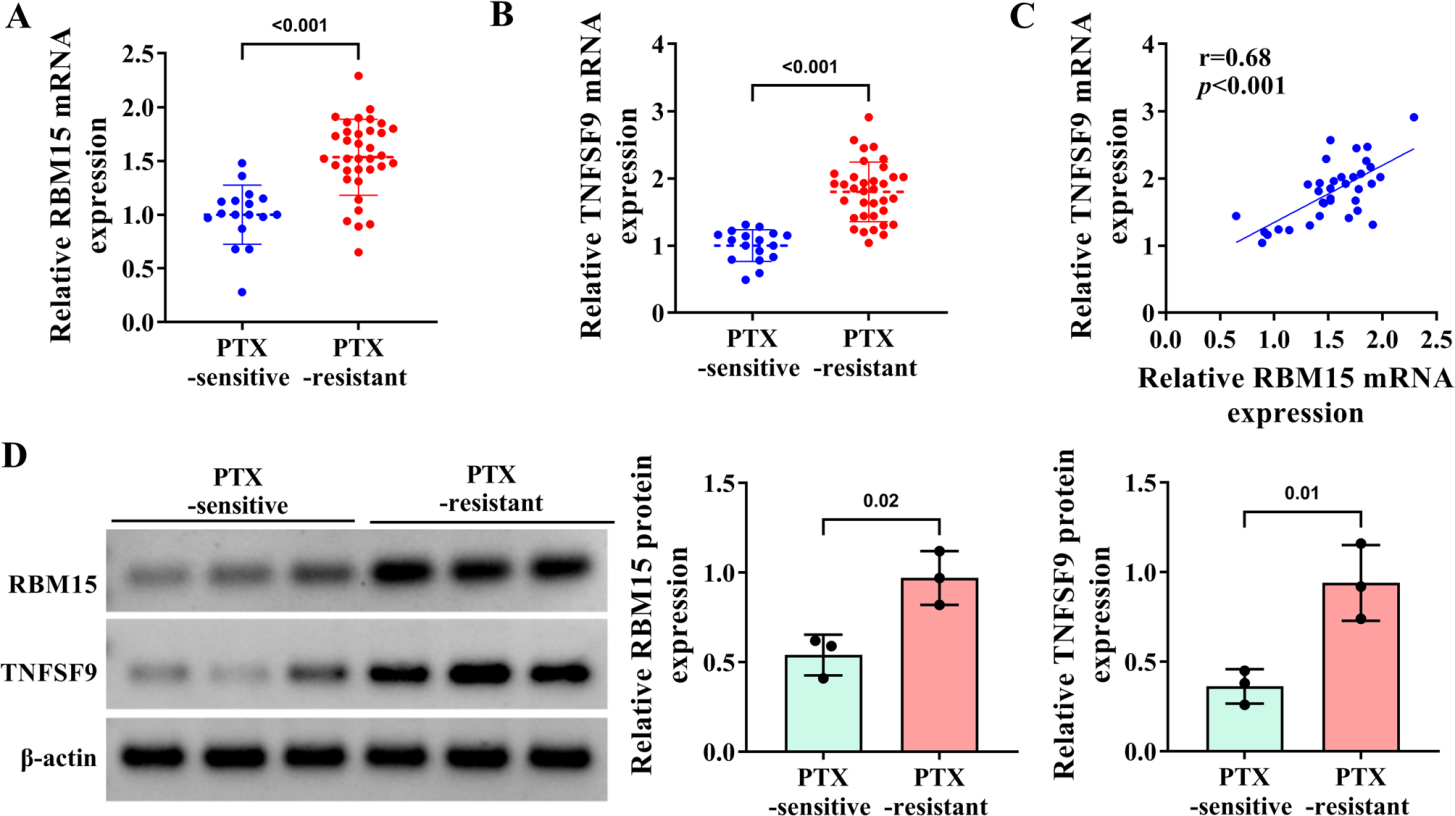
The expression of RBM15 and TNFSF9 in PTX-resistant TNBC patients. (**A**-**B**) RT-qPCR was used to detect the expression of RBM15 and TNFSF9 mRNA in tumor tissues of TNBC PTX-resistant (*n* = 35) and PTX-sensitive (*n* = 17) patients. (**C**) The correlation analysis of RBM15 and TNFSF9 expression in tumor tissues of TNBC PTX-resistant patients (*n* = 35). (**D**) The protein expression of RBM15 and TNFSF9 in tumor tissues of TNBC PTX-resistant and PTX-sensitive patients was detected by WB assay

Finally, using the PTX-sensitive group as a control, RT-qPCR analysis was performed on tumor tissues from TNBC patients with PTX resistance. The results revealed that either RBM15 or TNFSF9 mRNA were indeed highly expressed in the tumor tissues of PTX-resistant patients (Fig. 7A-B). Furthermore, the positive correlation between RBM15 and TNFSF9 in the tumor tissues of PTX-resistant patients was also proved by Spearman’s correlation analysis (Fig. 7C). On this basis, the preliminary conclusions of RT-qPCR were further verified by WB experiments. Undoubtedly, both RBM15 and TNFSF9 were conspicuously higher in the PTX-resistant group compared to the PTX-sensitive group (Fig. 7D). In summary, high expression of TNFSF9 and RBM15 played an important role in promoting the development of PTX resistance in TNBC patients.

## Discussion

TNBC, compared with other subtypes of breast cancer, carries a higher risk of recurrence, metastasis, and mortality, making it a particularly challenging type of breast cancer [27]. The absence of estrogen receptor, progesterone receptor, and HER-2 expression in TNBC makes traditional endocrine therapy and HER-2-targeted therapy ineffective. To date, chemotherapy remains the primary and almost exclusive option for TNBC treatment [28]. Unfortunately, most patients often develop chemotherapy resistance after a period of treatment, leading to rapid recurrence and progression of TNBC. This undoubtedly poses a major obstacle to TNBC treatment. The complex drug resistance mechanisms of TNBC have also been reported extensively in various studies in recent years [29]. Integrin β4, relying on the regulation of TNFAIP2 and IQGAP1, effectively activated RAC1, promoting the migration of TNBC cells while enhancing the resistance of cancer cells to DNA damage-related therapies [30]. Liu et al. confirmed that ALKBH5 promotes the characteristics of cancer stem cells in TNBC and enhances its resistance to doxorubicin by regulating the m6A methylation modification of FOXO1 mRNA [31]. Based on previous research, we conducted bioinformatics analysis to screen for the genes, which were upregulated in all five TNBC cell lines resistant to PTX. TNFSF9 among these genes has an agonistic effect on the development of various cancers and bone metastasis in breast cancer. Therefore, we then performed functional loss-of-function experiments on TNFSF9 to explore its impact on the resistance of TNBC cells to PTX. Obviously, the MDA-MB-231/PTX and MDA-MB-468/PTX cells with stable knockdown of TNFSF9 exhibited evidently enhanced sensitivity to PTX. Additionally, the silencing of TNFSF9 induced apoptosis in PTX-resistant TNBC cells, thereby strikingly repressed malignant functions as well as the glycolytic pathway. This discovery indicated that TNFSF9 is not only involved in the initiation and metastasis of TNBC, but also has a very close association with the resistance of TNBC to PTX.

It is well-established that macrophages play crucial roles in various physiological and pathological processes of the body, including defending against pathogens, regulating inflammation, and tissue repair [32, 33]. Furthermore, they are tightly linked to the onset and advancement of tumors. Specifically, the M1 phenotype is capable of recognizing, phagocytosing, and killing tumor cells, whereas the M2 phenotype induces tumor growth and metastasis [34]. According to reports, TNFSF9 regulated macrophage M2 polarization through the Src/FAK/p-AKT/IL-1β signaling pathway, thereby facilitating the worsening of pancreatic cancer [35]. Meanwhile, Han et al. reported that NF-κB, Wnt, and p53 pathways were upregulated in TNBC patients [12]. So, does TNFSF9 also participate in macrophage polarization in TNBC? We cultured THP1 cells using the supernatant of TNBC PTX-resistant cells with stable TNFSF9 knockdown. Flow cytometry results displayed a significant decrease in the expression of CD206, an M2 phenotype marker in THP1, while the expression of M1 phenotype iNOS was upregulated. It is more indirect that TNFSF9 may induce the polarization of macrophages to M2 type by promoting IL-10 and TGF-β secretion in TNBC cells. Anti-inflammatory cytokines secreted by M2 macrophages can inhibit immune responses and promote tumor progression. This indicated that silencing TNFSF9 induces the transition of M2 macrophages to M1. In other words, TNFSF9 regulated the M2 polarization of tumor-associated macrophages during TNBC progression. Similarly, RT-qPCR results for mRNA of downstream cytokine markers of the M1/M2 phenotypes also confirmed the above observation. Interestingly, by reviewing the literature, we found that tanshinone I inhibited late autophagy through the AKT/p38 MAPK signaling pathway, thereby enhancing chemotherapy sensitivity in TNBC [36]. Another report suggested that CXCR4 knockdown could mediate the PI3K/AKTmTOR pathway to enhance PTX resistance in ovarian cancer [37]. This suggests that the PTX resistance of TNBC is likely to be closely related to some downstream signaling pathways. Based on this, we hypothesized that in PTX-resistant TNBC cells, the high expression of TNFSF9 not only directly facilitates the metastatic process of TNBC but may also exacerbate the condition of TNBC by inducing macrophage polarization towards the M2 phenotype and expediting the release of cytokines.

There are over 100 modifications of RNA, among which m^6^A methylation is the most common form and a current research hotpot. As the name suggests, m^6^A RNA methylation refers to the chemical modification where a methyl group (-CH_3_) is added to the nitrogen atom N6 at the sixth carbon position of adenosine (A) [38]. It was the most widespread internal modification found in mammalian mRNA and holds a vital biological significance by modulating essential cellular functions. The m^6^A methyltransferases act as “writers” responsible for adding m^6^A modifications onto RNA molecules, ensuring the precision and efficiency of the methylation process. The key members of m^6^A methyltransferases primarily include METTL3, METTL14, WTAP and RBM15/RBM15B [39]. Notably, dysregulation of m^6^A modifications due to abnormal expression of regulatory proteins is often observed in many types of cancers [40, 41]. Ma MS and colleagues found that RBM15 -mediated m^6^A modification could stabilize the expression of RASSF8, thereby exacerbating the progression of lung adenocarcinoma cells [42]. RBM15 also regulated the IGF2BP1-YES1-MAPK signaling axis through the m6A modification mechanism and accelerated the progression of hepatocellular carcinoma [43]. In addition, relevant reports have also indicated that the m^6^A methyltransferase RBM15 enhanced the proliferation of TNBC cells by accelerating the metabolic process of serine and glycine [18]. Referring to these conclusions, we speculate that RBM15 may enhance the resistance of TNBC to PTX through m^6^A modification. Through screening on the Linkedomics website, we found that RBM15 has a certain positive correlation with TNFSF9, which is our focus. The SARMP website showed that TNFSF9 had an m6A site in its mRNA sequences, and the RBPsuite website predicted that TNFSF9 had potential binding sites with RBM15. Meanwhile, it was confirmed that RBM15 was also highly expressed in TNBC PTX-resistant cells as was TNFSF9. And in PTX-resistant TNBC cells with stable knockdown of RBM15, the expression of TNFSF9 was also down-regulated. As we predicted, RBM15 could bind to TNFSF9 and regulate its mRNA m^6^A modification, a conclusion that has been verified through RIP and MeRIP experiments. Of particular note, our research revealed that after actinomycin treatment, the expression level of TNFSF9 mRNA in the sh-RBM15 cell line robustly decreased, which strongly demonstrated the destabilizing effect of RBM15 silencing on TNFSF9 mRNA. Subsequently, to further validate the interplay between RBM15 and TNFSF9, we performed a rescue experiment involving the silencing of RBM15. Interestingly, we observed that the inhibition of proliferation, migration, invasion, and glycolysis in MDA-MB-231 and MDA-MB-468 cells originally caused by RBM15 silenced was completely eliminated in the presence of TNFSF9 overexpression. At the same time, tumor-associated macrophages that originally tended to adopt the M1 phenotype shifted more toward the M2 phenotype. Furthermore, the mRNA expression of M2 phenotype-related cytokines IL-10 and TGF-β were re-upregulated. These phenomena indicated that the high sensitivity of TNBC cells to PTX resulting from RBM15 knockdown is disrupted by TNFSF9 overexpression. In short, RBM15 exerts additional influence on the drug resistance of TNBC cells to PTX by modulating the expression levels of TNFSF9.

More crucially, we have clearly demonstrated through the use of mice TNBC tumor allograft model experiment that silencing RBM15 could greatly block tumor growth in mice. However, when TNFSF9 was overexpressed, the therapeutic effect of PTX on the tumor was obviously reduced. What’s more, through WB analysis of tumor tissues, we further verified the existence of a clear positive correlation between TNFSF9 and RBM15 in tumors. Ultimately, to validate the clinical applicability of our conclusions, we collected tumor tissue samples from TNBC patients who were sensitive and resistant to PTX. The results clarified that, compared to PTX-sensitive patients, the expression levels of RBM15 and TNFSF9 were abnormally high in the tumor tissues of PTX-resistant patients, and there was a relatively significant positive correlation between the two. Additionally, we conducted a WB assay on the patients’ tumor tissues, which also indicated that the expression of RBM15 and TNFSF9 were drastically upregulated in the PTX-resistant in contrast to the PTX-sensitive group. This suggested that TNFSF9 and RBM15 are likely to be key factors contributing to the resistance of TNBC patients to PTX.

In summary, this study has unveiled an important finding: RBM15 may exacerbate the resistance of TNBC to PTX and accelerate the progression of TNBC by regulating the m6A methylation modification of TNFSF9 mRNA and inducing M2 polarization of TNBC tumor-associated macrophages. Therefore, our research not only delves into the mechanisms of TNBC drug resistance but also provides new potential targets for TNBC treatment strategies. Unfortunately, although the study revealed important mechanisms, several limitations remain. Research has focused on RBM15-induced polarization of macrophages to M2 type, but macrophage polarization is a complex and dynamic process that may involve multiple signaling pathways and cytokines. In the future, the effects of other factors (such as cytokine network and metabolic state) on macrophage polarization need to be fully explored. Meanwhile, the overexpression of ATP-binding cassette transporters such as P-gp is the classical mechanism of PTX resistance, and its expression level in RBM15 high expression cells needs to be detected. More importantly, TNBC is highly heterogeneous, and different molecular subtypes differ significantly in their sensitivity to PTX. Even if RBM15 is highly expressed in a subset of patients, its interaction with TNFSF9 may only apply to specific subsets and needs to be further stratified by single-cell sequencing or spatial transcriptomics.

## Supplementary Information

Below is the link to the electronic supplementary material.

Supplementary Material 1

Supplementary Material 2

## Data Availability

No datasets were generated or analysed during the current study.
