# Supplementary material for: RBM15 enhances paclitaxel resistance in triple-negative breast cancer by targeting m6A methylation of TNFSF9 and inducing polarization of tumor-associated macrophages to M2 phenotype
Source: Hereditas. 2025 Aug 19;162:167. doi: 10.1186/s41065-025-00534-0 (PMC12362948; doi:10.1186/s41065-025-00534-0)

**Supplementary Figure 1 Determination of M1 (iNOS) and M2 (CD206) marker expression levels in tumor tissues of PTX-sensitive and PTX-resistant patients.** (A-B) The mRNA levels of CD206 and iNOS in tumor tissues of PTX-sensitive and PTX-resistant patients were monitored by RT-qPCR.


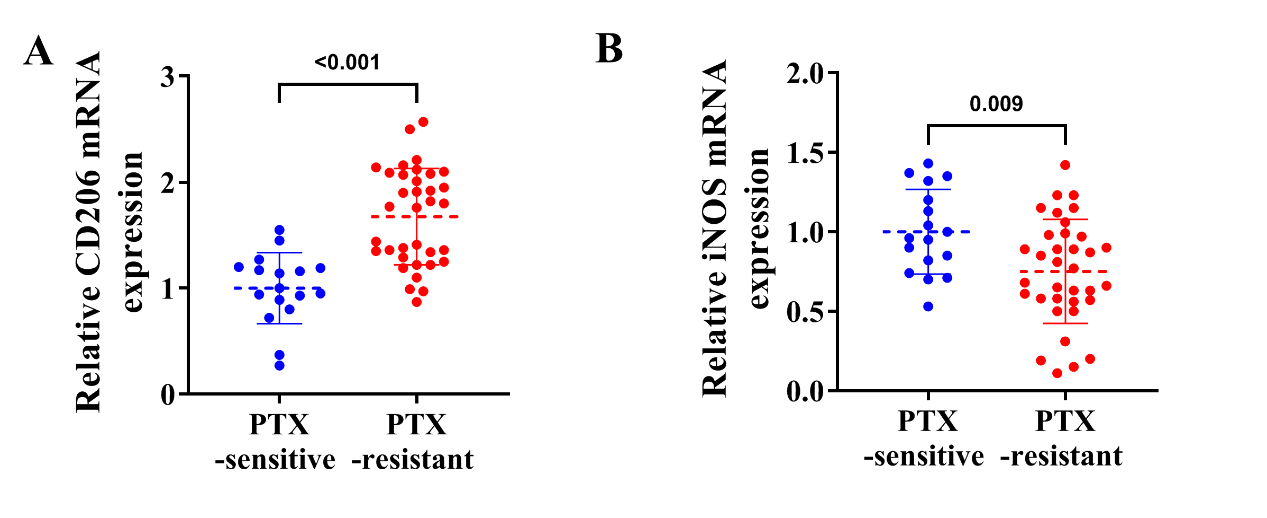

Supplement: Supplementary file 1 — Supplementary Material 1 [file 41065_2025_534_MOESM1_ESM.docx]
