# Supplementary material for: RBM15 enhances paclitaxel resistance in triple-negative breast cancer by targeting m6A methylation of TNFSF9 and inducing polarization of tumor-associated macrophages to M2 phenotype
Source: Hereditas. 2025 Aug 19;162:167. doi: 10.1186/s41065-025-00534-0 (PMC12362948; doi:10.1186/s41065-025-00534-0)
Supplement: Supplementary file 2 — Supplementary Material 2 [file 41065_2025_534_MOESM2_ESM.pdf]

Fig 1I

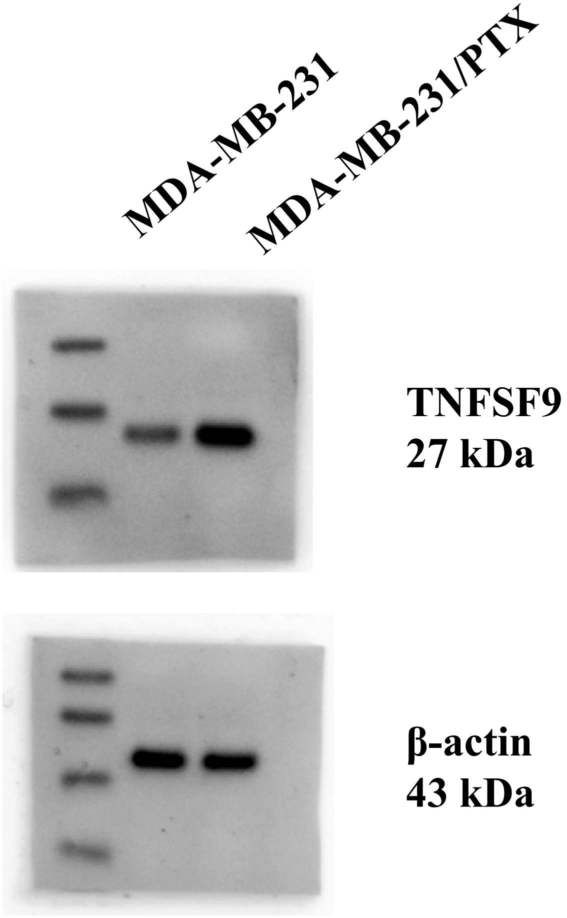
MDA-MB-231
MDA-MB-231/PTX
TNFSF9
27 kDa
β-actin
43 kDa

Fig 1J

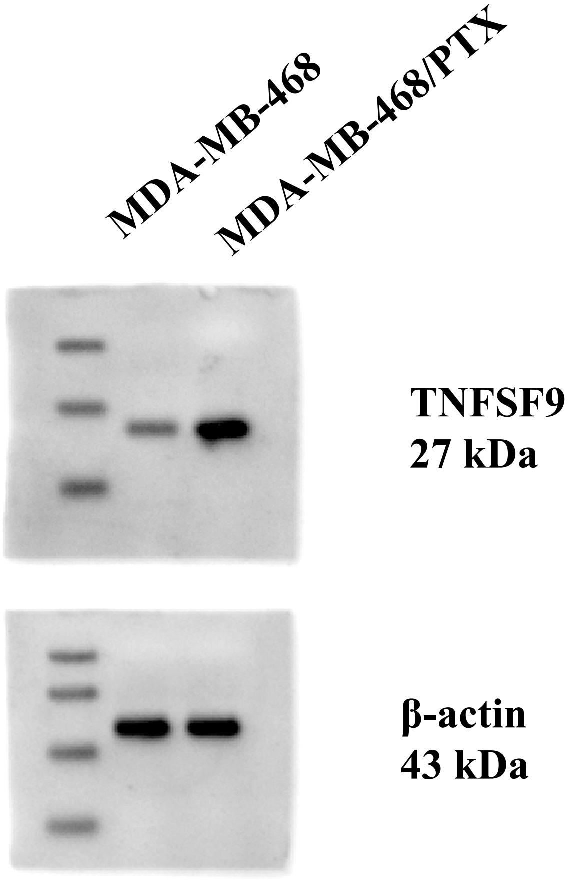
MDA-MB-468
MDA-MB-468/PTX
TNFSF9
27 kDa
β-actin
43 kDa

Fig 2A

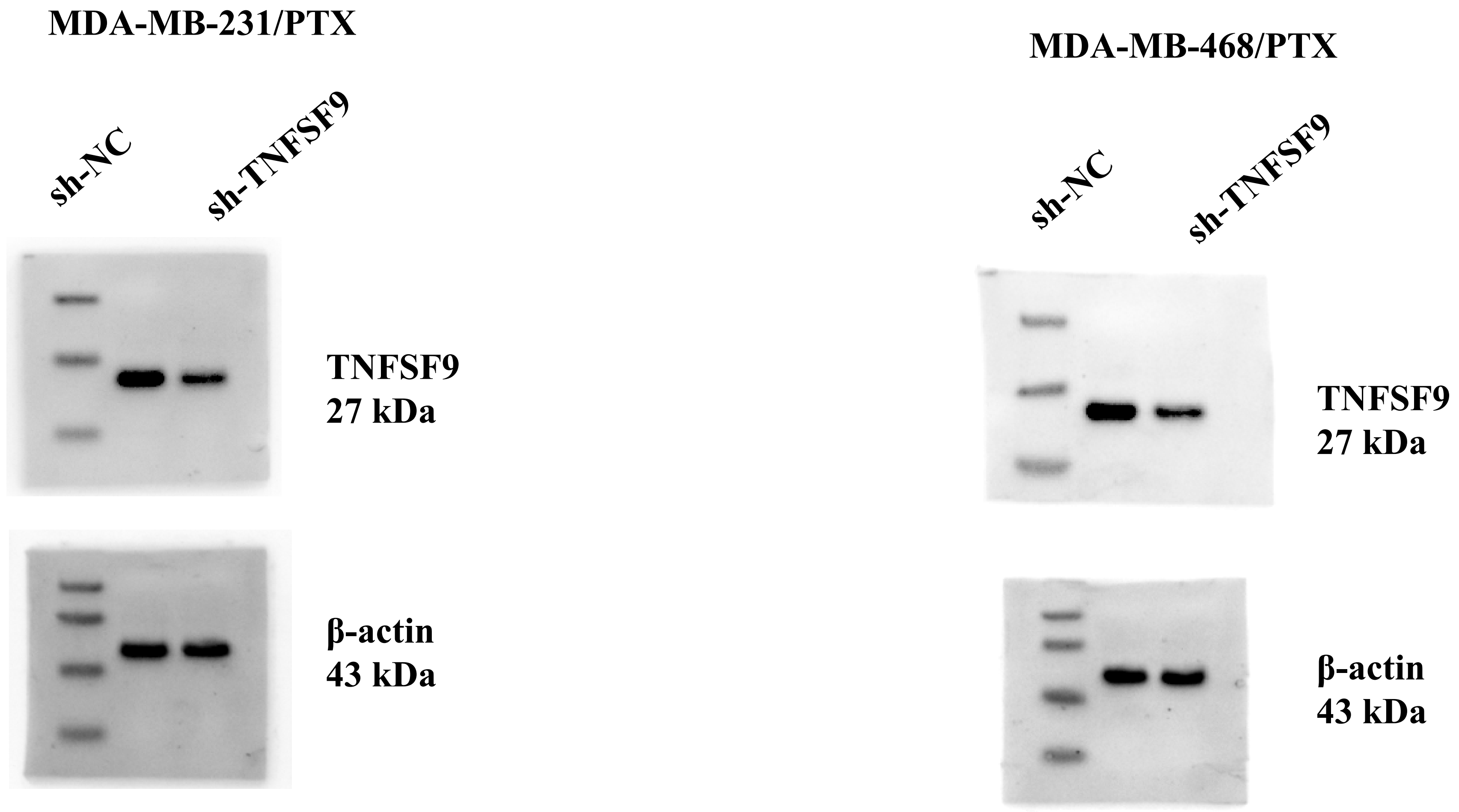
MDA-MB-231/PTX
sh-NC
sh-TNFSF9
TNFSF9
27 kDa
β-actin
43 kDa
MDA-MB-468/PTX
sh-NC
sh-TNFSF9
TNFSF9
27 kDa
β-actin
43 kDa

Fig 4G

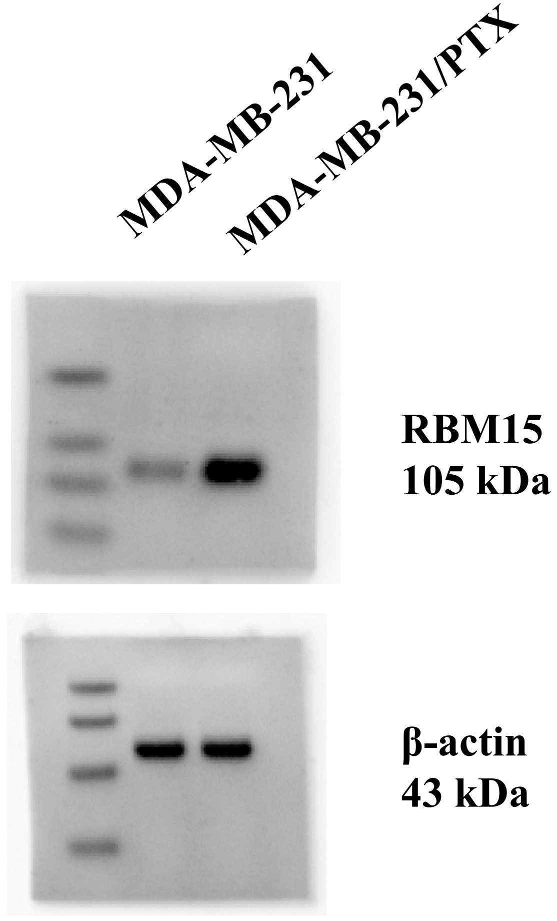
MDA-MB-231
MDA-MB-231/PTX
RBM15
105 kDa
β-actin
43 kDa

Fig 4H

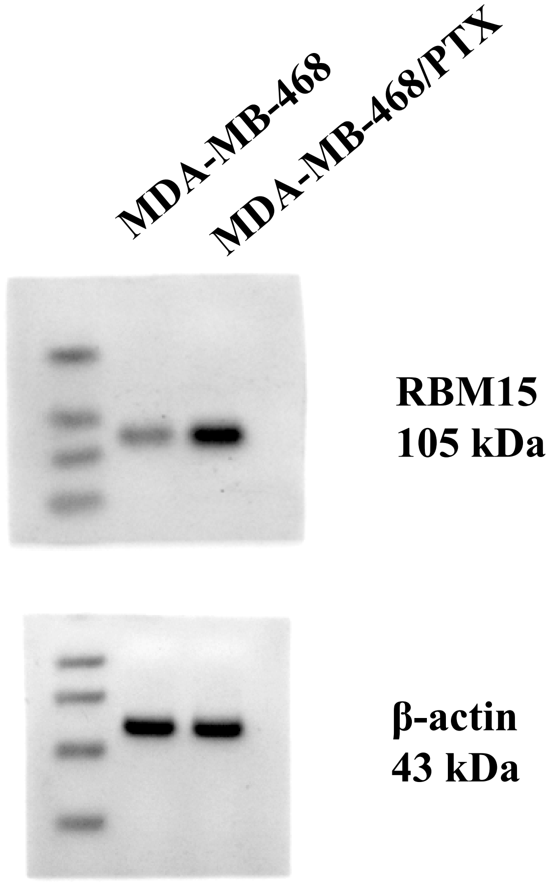
MDA-MB-468
MDA-MB-468/PTX
RBM15
105 kDa
β-actin
43 kDa

Fig 4I

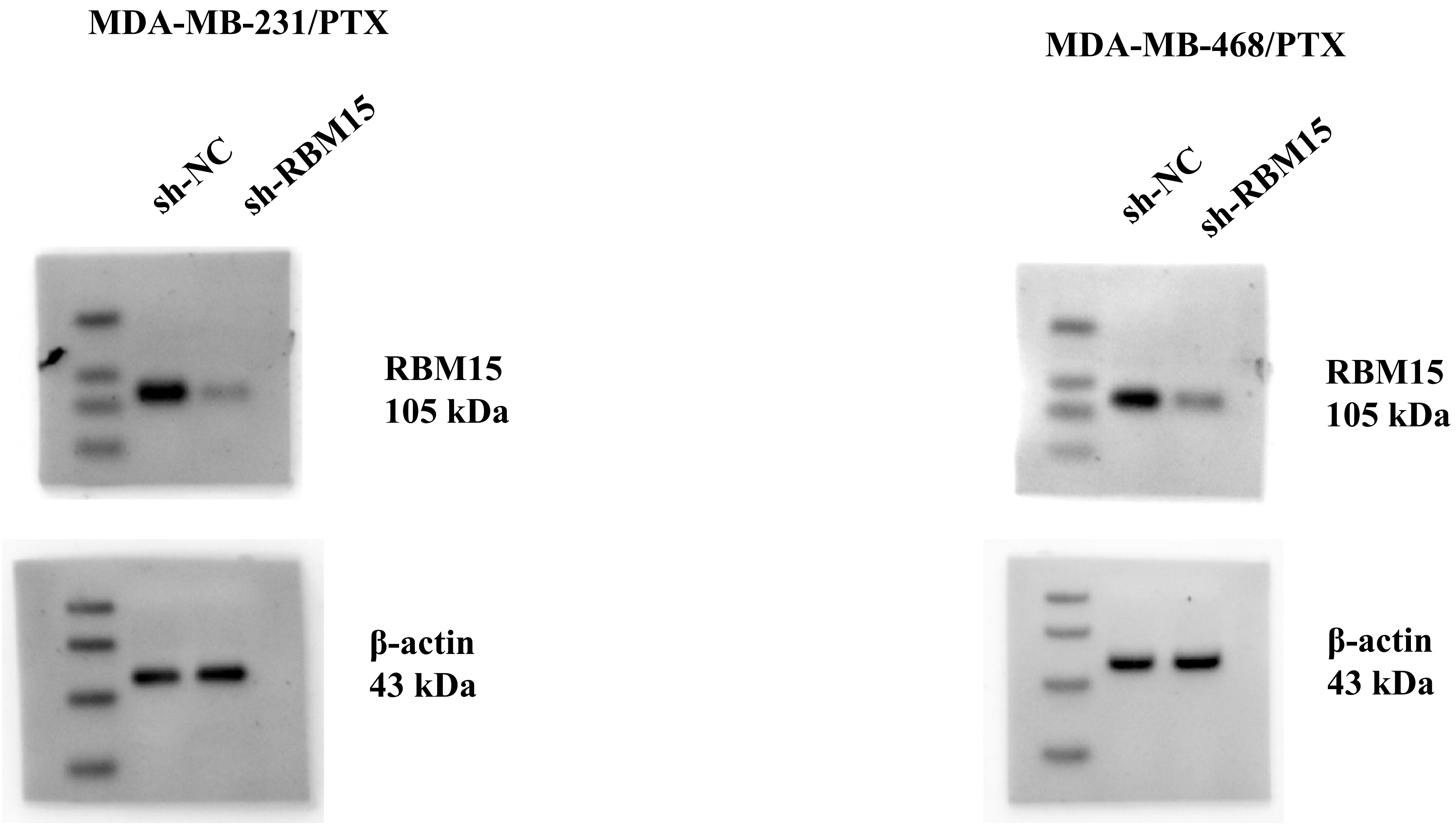
MDA-MB-231/PTX
sh-NC
sh-RBM15
RBM15
105 kDa
β-actin
43 kDa
MDA-MB-468/PTX
sh-NC
sh-RBM15
RBM15
105 kDa
β-actin
43 kDa

Fig 4K

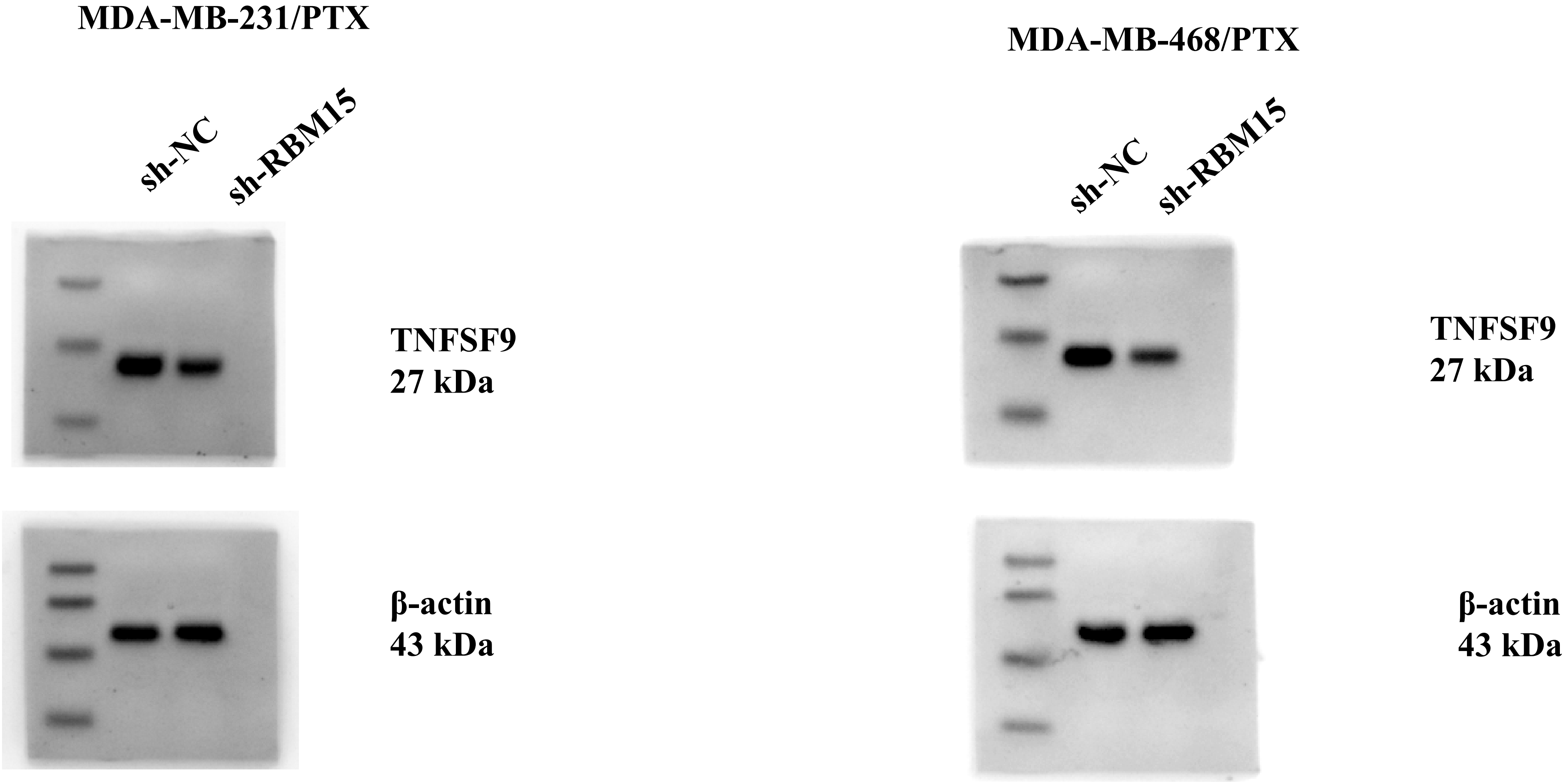
MDA-MB-231/PTX
sh-NC
sh-RBM15
TNFSF9
27 kDa
β-actin
43 kDa
MDA-MB-468/PTX
sh-NC
sh-RBM15
TNFSF9
27 kDa
β-actin
43 kDa

Fig 5A

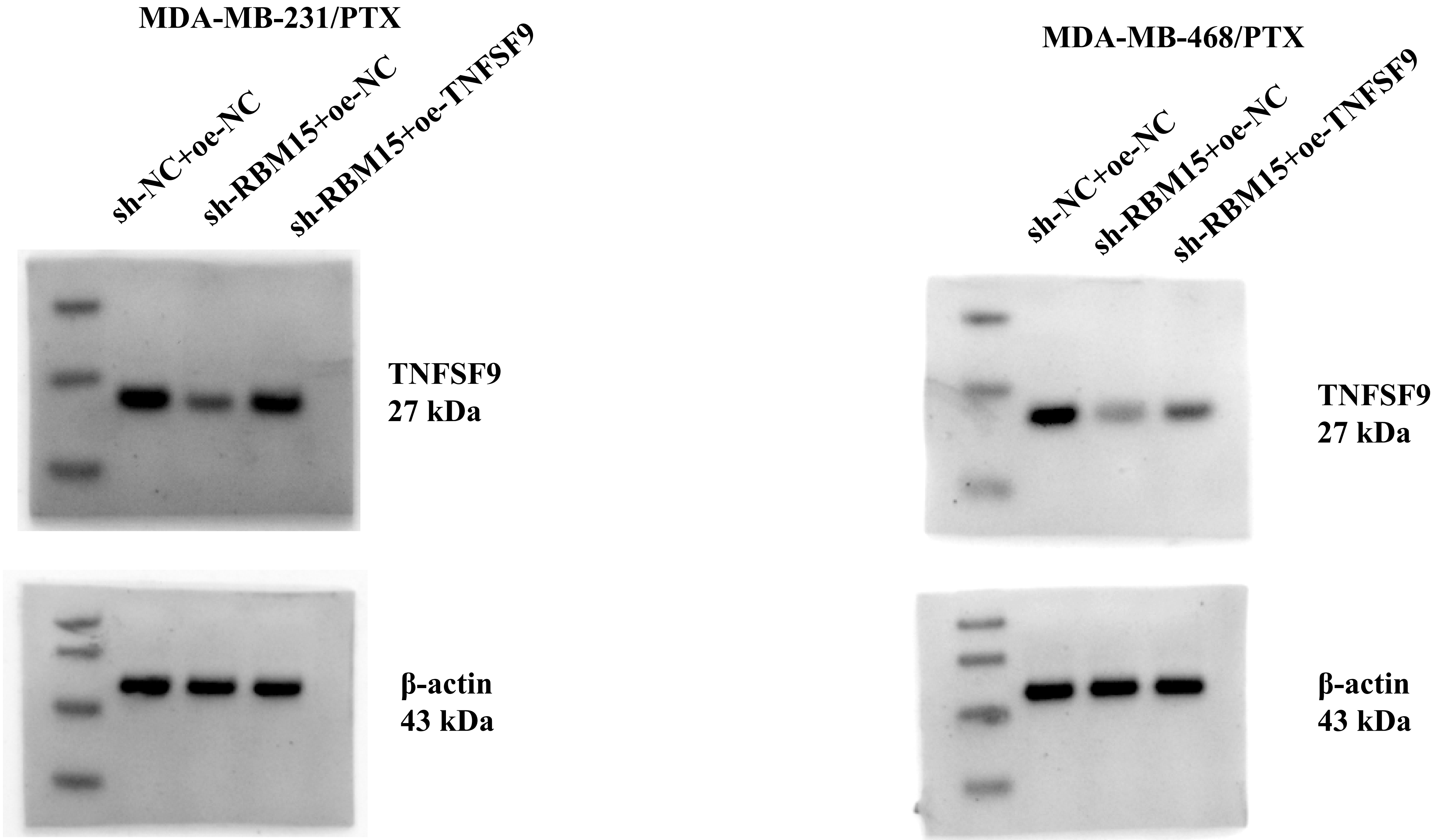
MDA-MB-231/PTX
sh-NC+oe-NC
sh-RBM15+oe-NC
sh-RBM15+oe-TNFSF9
TNFSF9
27 kDa
β-actin
43 kDa
MDA-MB-468/PTX
sh-NC+oe-NC
sh-RBM15+oe-NC
sh-RBM15+oe-TNFSF9
TNFSF9
27 kDa
β-actin
43 kDa

Fig 6C

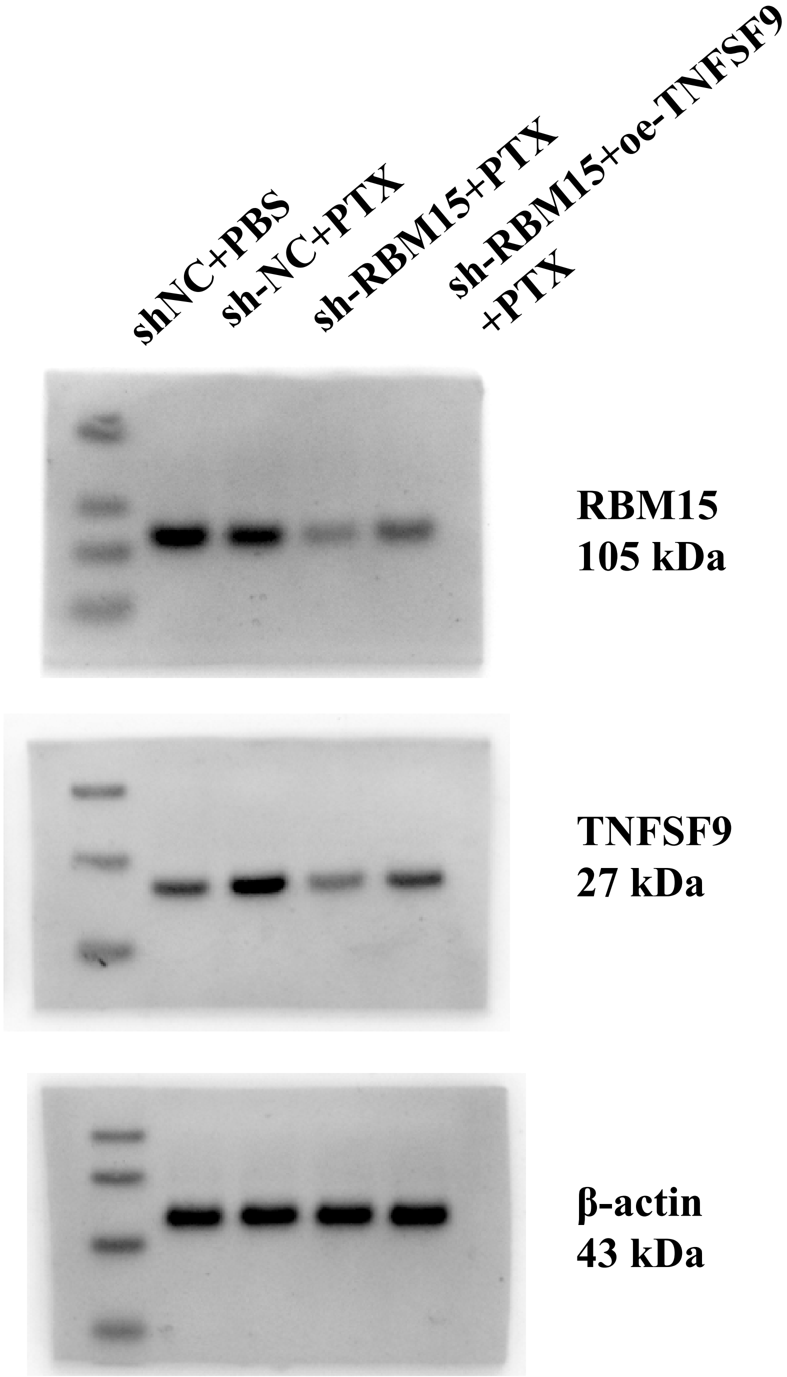
shNC+PBS
sh-NC+PTX
sh-RBM15+PTX
sh-RBM15+oe-TNFSF9
+PTX
RBM15
105 kDa
TNFSF9
27 kDa
β-actin
43 kDa

Fig 7A

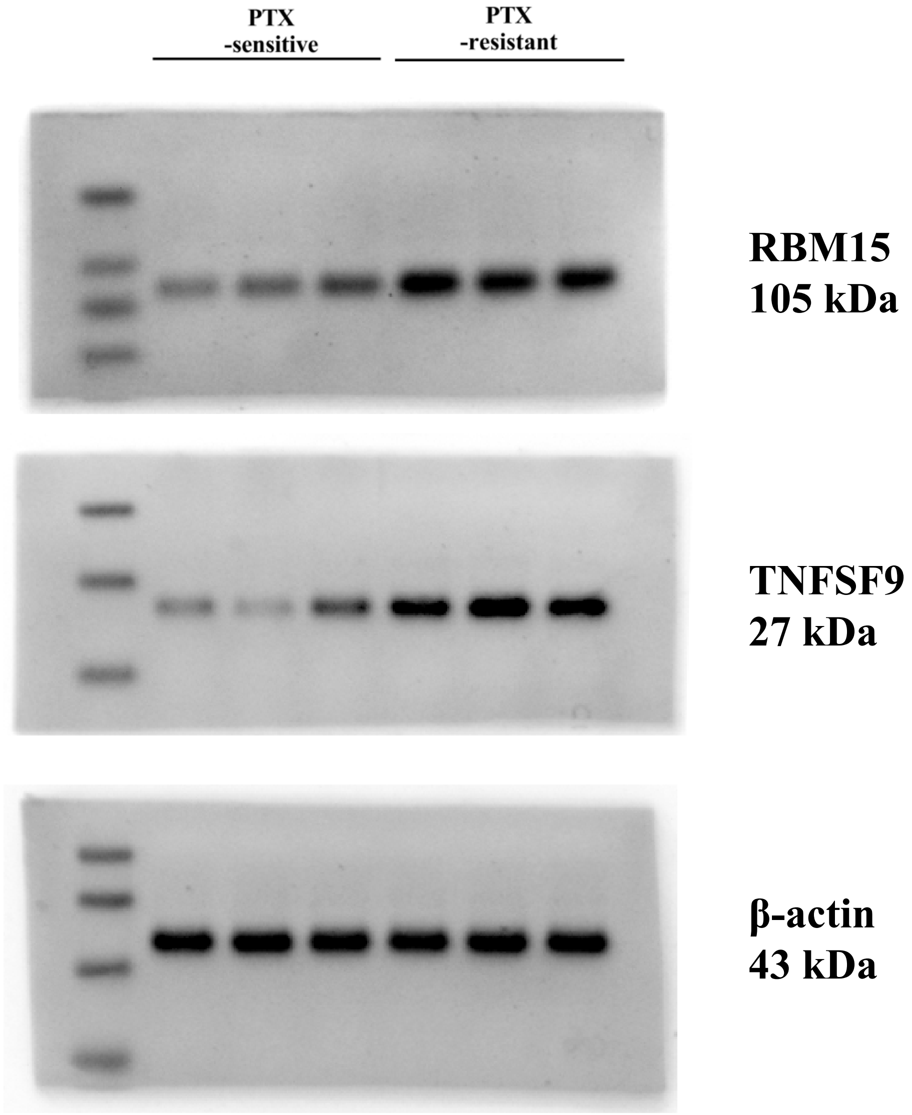
PTX
-sensitive
PTX
-resistant
RBM15
105 kDa
TNFSF9
27 kDa
β-actin
43 kDa
